# Epidermal growth factor receptor mutation mediates cross-resistance to panitumumab and cetuximab in gastrointestinal cancer

**DOI:** 10.18632/oncotarget.3574

**Published:** 2015-03-14

**Authors:** Friederike Braig, Manuela März, Aneta Schieferdecker, Alexander Schulte, Mareike Voigt, Alexander Stein, Tobias Grob, Malik Alawi, Daniela Indenbirken, Malte Kriegs, Erik Engel, Udo Vanhoefer, Adam Grundhoff, Sonja Loges, Kristoffer Riecken, Boris Fehse, Carsten Bokemeyer, Mascha Binder

**Affiliations:** ^1^ Department of Oncology and Hematology, BMT with section Pneumology, Hubertus Wald Tumorzentrum / UCCH, University Medical Center Hamburg-Eppendorf, Hamburg, Germany; ^2^ Department of Neurosurgery, Laboratory for Brain Tumor Biology, University Medical Center Hamburg-Eppendorf, Hamburg, Germany; ^3^ Department of Pathology, University Medical Center Hamburg-Eppendorf, Hamburg, Germany; ^4^ Bioinformatics Service Facility, University Medical Center Hamburg-Eppendorf, Hamburg, Germany; ^5^ Heinrich-Pette-Institute, Leibniz-Institute for Experimental Virology (HPI), Hamburg, Germany; ^6^ Radiation Biology and Radio-Oncology, University Medical Center Hamburg-Eppendorf, Hamburg, Germany; ^7^ Hämatologisch-onkologische Praxis Altona (HOPA), Hamburg, Germany; ^8^ Marienkrankenhaus, Zentrum für Innere Medizin, Hamburg, Germany; ^9^ Institute for Tumor Biology, University Medical Center Hamburg-Eppendorf, Hamburg, Germany; ^10^ Research Department Cell and Gene Therapy, Department of Stem Cell Transplantation, University Medical Center Hamburg-Eppendorf, Hamburg, Germany

**Keywords:** panitumumab, cetuximab, EGFR antibody resistance, mutation, circulating tumor DNA

## Abstract

Acquired resistance to epidermal growth factor receptor (EGFR) targeted antibodies represents a clinical challenge in the treatment of gastrointestinal tumors such as metastatic colorectal cancer, but its molecular mechanisms are incompletely understood. We scanned KRAS exon 2/3/4, NRAS exon 2/3/4 and the overlapping epitopes of the EGFR antibodies cetuximab and panitumumab for mutations in pre- and post-treatment tumor tissue of 21 patients with gastrointestinal cancer treated with chemotherapy +/− EGFR antibodies by next-generation sequencing (“tumor tissue” cohort). We describe a novel EGFR exon 12 mutation acquired in tumors of 1 out of 3 patients treated with panitumumab. The EGFR G465R mutation introduces a positive charge within the overlap of the panitumumab and cetuximab epitopes. It abrogates antibody binding and mediates cross-resistance to both antibodies in EGFR G465R-transfected Ba/F3 cells. In circulating tumor DNA from an independent “liquid biopsy” cohort of 27 patients, we found this novel mutation in 1 out of 6 panitumumab-treated cases while about one third of patients show acquired RAS mutations. We show that acquired resistance by epitope-changing mutations also emerges during panitumumab treatment, which can be easily detected by a liquid biopsy approach even before clinical resistance occurs and this may help in tailoring EGFR-targeted therapies.

## INTRODUCTION

Monoclonal antibodies which inhibit downstream pathway signaling by targeting the extracellular ligand binding domain have become one of the mainstays of EGFR inhibition. For the treatment of metastatic colorectal cancer (mCRC) the chimeric EGFR antibody cetuximab and the fully human antibody panitumumab were approved as single agents or in combination with chemotherapy [1-11]. Both antibodies were also used in patients with gastric or pancreatic cancer, cholangiocellular carcinoma (CCC) or other gastrointestinal cancers in clinical trials [12-17]. Resistance to these antibodies is mediated by mutations in downstream signaling molecules [18-21], with mutated *RAS,* which is currently the only validated and widely accepted molecular marker that predicts lack of response to EGFR antibodies and, therefore, guides treatment decisions in mCRC [20, 22-25]. Therefore, patients are routinely screened for *KRAS* exon 2/3/4 and *NRAS* exon2/3/4 mutations before the initiation of EGFR targeted therapy [26, 27]. However, even patients without *RAS* mutations who primarily respond well to EGFR antibodies will eventually develop secondary resistance limiting the clinical benefit of these drugs.

Some recent studies have addressed the molecular mechanisms underlying acquired resistance. Accumulating evidence suggests that *RAS* wt tested tumors may harbor small *RAS* mutated subclones at diagnosis that emerge and thus mediate secondary resistance under the selective pressure of treatment with EGFR antibodies [28-30]. Moreover, very recently a mutation in the ectodomain of *EGFR* leading to the substitution of serine by arginine in position 492 has been described. This mutation can be acquired during therapy with cetuximab and mediates resistance to this antibody (but not to panitumumab) by abrogating its binding to the EGFR [31, 32]. Differential resistance in this mutant is not surprising as we could recently show that the large conformational EGFR domain III epitopes of both antibodies only partially overlap and position S492 belongs exclusively to the cetuximab binding site [33].

Here, we investigated *EGFR* ectodomain and *RAS* mutations in patients with gastrointestinal cancer treated with EGFR-targeting antibodies and describe for the first time a panitumumab-induced EGFR mutation that mediates cross-resistance to both panitumumab and cetuximab by critically changing an amino acid position localized within the overlap of both antibody epitopes. Perspectively, screening of ctDNA for EGFR ectodomain mutations may be helpful in monitoring patients for resistance-mediating tumor subclones.

## RESULTS

### Clinical characteristics of the “tumor tissue” patient cohort

16 EGFR antibody-naïve patients of the “tumor tissue” patient cohort were treated with cetuximab or panitumumab in combination with chemotherapy as shown in Table 1. EGFR antibodies were applied after an average of one prior therapy and the majority of patients showed at least stable disease. The mean duration of EGFR antibody treatment prior to secondary surgery and thus post-treatment sample acquisition was 4.8 months. Five patients treated with the VEGF antibody bevacizumab in combination with chemotherapy were used as control group.

**Table 1:** Clinical characteristics of the "tumor tissue" patient cohort.*

pat. #	category	age [years)	sex	tumor site	stage (AJCC/TNM)	KRAS status	no. of previous treatments	treatment	treatment duration [months]	best response
1	panitumumab group	36	rn	rectum	lit	wt	1	Foil oxiPan	3	PD
2		50	m	colon	IV	wt	0	Folt ox/Pan	3	PR
3		70	m	rectum	IV	wt	1	Foil ki/Pan	8	PR
4		59	m	colon	IV	wt	3	FothriPan	3	PD
5		46	f	colon	IV	wt	3	5•FurFsvPan	6	SD
6		62	m	CCC	IV	wt	0	Ce/GemPan	6	SD
7		75	rn	CCC	IV	wt	0	Gs/Geer/Pan	6	SD
8		62	m	rectum	IV	wt	1	Follii/Pan	6	PR
9	cetuximab group	76	*in*	rectum	IV	wt	1	FollwieCet	3	SD
10		70	f	colon	IV	wt	1	Fortin/eat	6	SD
11		68	m	rectum	1118	wt	1	Met/SM	6	PR
12		58	m	rectum	IV	wt	1	InCet	3	PR
13		59	rn	rectum	IV	wt	2	Folf ii/Cet	3	PR
14		43	rn	rectum	IV	wt	1	FothriCet	6	SD
15		47	f	CUP	IV	wt	0	Cato/Fact/Cet	4	PR
16		72	in	CUP	IV	wt	0	Carbo/Fact/Cet	4	PD
17	control group	74	f	rectum	IV	mut.	0	5•1.1/FA/Bev	3	SD
18		53	m	colon	IV	wt	0	XeloxBev	3	PR
19		58	in	rectum	IV	mut.	0	FollowyBev	4	PR
20		65	rn	colon	IV	mut.	0	Xelox/Bev. Xelox	2/6	PD
21		58	m	colon	IV	wt	0	Fotrousev	2	SD

* Stage refers to the stage at diagnosis; treatment, treatment duration and response refers to the indicated antibody-containing treatment or the control treatment. respectively; KRAS status was established by routine clinical testing at diagnosis covering exon 2 and 3 mutations, response was evaluated according to recist criteria. Bev= Bewizumab, Carbo = carboplatinum, Cet = Cetuximab. Cis = cisplatinum, FA = blinic acid, Gem = gemcitabine, Iri = irinotecan, Pacli = paclitaxel, Pan = Panitumumab, SM = study medication. Xelox = Yeloda + oxaliplatinum, 5•FU = 5•ffuorouracile: CCC = cholangiocellular cancer. CUP = cancer of unknown primary: CR = complete remission, PR = partial remission, SD = stable disease. PD = progressive disease

### Targeted *NGS* of *EGFR* and *RAS* in samples from the “tumor tissue” cohort

*KRAS* 2/3 status of baseline samples (determined by routine clinical testing) was confirmed by targeted NGS of these exons. In addition, the mutational status of *KRAS* exon 4 and *NRAS* exon 2/3/4 was determined by NGS at baseline (Table 2). Interestingly, tissue samples from patients tested as *RAS* wt at baseline showed no evidence for *RAS* mutated minimal subclones after treatment.

**Table 2:** NGS of EGFR axons 7-13, KRAS exons 2/3/4 and NRAS exons 2/3/4 in pre- and post-treatment samples from the "tumor tissue" patient cohort.*

		time point 1				time point 2			
pat. #	category	sample 1	EGFR (ex.7-13)	KRAS (ex. 2/3/4)	NRAS (ex. 2/3/4)	sample 2	EGFR (ex. 7-13)	KRAS (ex. 2/3/4)	NRAS (ex. 2/3/4)
1	panitumumab group	primary tumor	wt	wt	wt	liver metastasis	wt	wt	wt
2		primary tumor	wt	wt	wt	primary tumor/LN1/LN2	G465R (3.5%)/G465R (6.8%)/wt	wt/wt/wt	wt/wt/wt
3		primary tumor	wt	wt	wt	-	-	-	-
4		liver metastasis	wt	wt	wt	-	-	-	-
5		-	-	-	-	lung metastasis	wt	wt	wt
6		liver metastasis	wt	wt	wt	-	-	-	-
7		liver metastasis	wt	wt	cod. 61 (Q-> K. 22.2%)	-	-	-	-
8		primary tumor	wt	wt	wt	-	-	-	-
9	cetuximab group	-	-	-	-	liver metastasis	wt	wt	wt
10		primary tumor	wt	wt	wt	-	-	-	-
11		-	-	-	-	primary tumor	wt	wt	wt
12		liver metastasis	wt	wt	wt	-	-	-	-
13		liver metastasis	wt	wt	wt	liver metastasis	wt	wt	wt
14		-	-	-	-	abdominal metastasis	wt	wt	wt
15		LN metastasis	wt	wt	wt	-	-	-	-
16		primary tumor	wt	wt	wt	-	-	-	-
17	control group (w/o EGFR-targeted therapy)	primary tumor	wt	cod. 12 (G -> V. 20.4%)	wt	liver metastasis	wt	wt	wt
18		-	-	-	-	liver metastasis	wt	wt	wt
19		-	-	-	-	liver metastasis	wt	cod. 12 (G->V, 38.7%)	wt
20		liver metastasis	wt	cod. 12 (G->D, 40.2%)	wt	-	-	-	-
21		primary tumor	wt	wt	wt	liver metastasis	wt	wt	wt

* time point 1 = pre-treatment (rfering to panitumumab/cetuximab/control treatment), time point 2 = post-treatment (refering to panitumumab/cetuximab/control treatment); wt = wild type, LN = lymph node, ex. = exon, cod.= codon, % = percentage of reads

**Figure 1:**
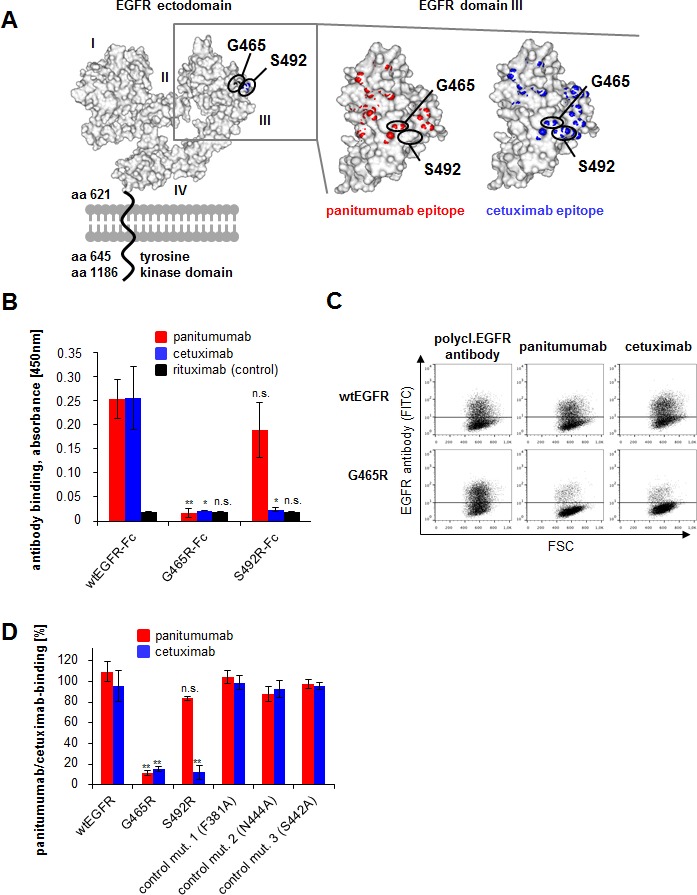
*EGFR G465R* mutant reveals almost complete abrogation of panitumumab and cetuximab binding A: Localization of *EGFR* mutations *G465R* and *S492R* on the ectodomain of the *EGFR*. The 3-dimensional EGFR model was created from pdb file 1NQL from RCSB Protein Data Bank. The S492 position is shown in blue, the G465 position in black. The panel on the right shows the EGFR domain III alone with panitumumab epitope in red and cetuximab epitope in blue. Overlaps of mutated positions G465 and S492 with antibody epitopes are shown. B: *EGFR* mutation *G465R* abrogates binding of panitumumab and cetuximab at the protein level. Wile-type and mutant EGFR-Fc proteins were expressed and the binding of therapeutic antibodies to immobilized proteins was assessed by ELISA. Data are means from 3 experiments +/− SEM. * p<0.05, ** p<0.01, n.s. = not significant (student's T-test comparing binding of respective antibody to mutant versus wt EGFR-Fc) C and D: Panitumumab and cetuximab binding is abrogated in CHO cells transfected with *EGFR G465R*. *EGFR* negative CHO cells were transfected with wild type *EGFR* or mutants thereof. Binding of panitumumab, cetuximab or a control polyclonal *EGFR* antibody was assessed by FACS analysis 48h after transfection. FSC = forward scatter. Panel C shows exemplary FACS plots, panel D shows mean data from 5 experiments with binding of the polyclonal *EGFR* antibody set to 100% +/− SEM. * p<0.05, ** p<0.01, n.s. = not significant (student's T-test comparing binding of respective antibody to mutant versus wt EGFR).

In addition, we performed NGS to identify mutations in the *EGFR* ectodomain potentially interfering with antibody binding. None of the pre-treatment or control samples showed *EGFR* ectodomain mutations in exons 7-13. In 1 out of 3 patients treated with panitumumab we found an acquired *EGFR G465R* ectodomain mutation after treatment with panitumumab and FOLFOX in post-treatment tumor material (patient 2, Table 2). This novel exon 12 mutation was localized in proximity to the previously described cetuximab-induced *S492R* mutation (Figure 1A) [31]. It constituted 3.5% of all exon 12 reads and could also be detected in a tumor-infiltrated lymph node resected together with the local tumor after treatment with panitumumab and FOLFOX at a frequency of 6.8%. Deep sequencing of pre-treatment primary tumor (Table 2) as well as peripheral blood leukocyte DNA from this patient resulted in 100% germline sequence for exon 12 (data not shown) confirming the acquired nature of the mutation.

### Binding profile of *EGFR* mutation *G465R*

Based on structure analysis position, G465 is located right in the center of the overlap region of the large conformational cetuximab and panitumumab epitopes previously characterized by our group (Figure 1A). We therefore hypothesized that the introduction of a positive charge at this position due to the glycine to arginine exchange in the malignant cells may abrogate not only panitumumab, but also cetuximab binding. To address this question, we generated a recombinant *EGFR* variant containing the same amino acid substitution (glycine to arginine: G465R). The mutation was introduced by site-directed mutagenesis in a vector suitable for protein expression of the EGFR ectodomain as Fc-fusion protein (EGFR-Fc) and a vector suitable for membrane expression of the whole receptor. Furthermore, the previously published *EGFR S492R* mutant was included in the experiment. Correct expression and immobilization of EGFR-Fc wt and mutant proteins was assessed by ELISA (Supplementary Figure 2). Whilst panitumumab did not bind to immobilized EGFR-Fc G465R protein, its binding to wt EGFR-Fc and EGFR-Fc S492R was preserved (Figure 1B). Cetuximab only bound to wt EGFR-Fc, but to neither of the mutant receptors (Figure 1B). Moreover, EGFR wt and mutant constructs were transfected into EGFR-negative CHO cells and binding of panitumumab and cetuximab to membrane-expressed wt and mutant receptors was analyzed by flow cytometry (Figure 1C and D). A polyclonal EGFR antibody served as a control for receptor expression. As previously shown, cetuximab binding was significantly inhibited in the *EGFR S492R* mutant, while panitumumab binding was preserved. In the *EGFR G465R* mutant, binding of both antibodies was almost completely abrogated (binding reduction of 85-90%), in concordance with the central localization of the mutation within the epitopes of both antibodies.

### Functional validation of *EGFR* mutation *G465R* in Ba/F3 cells

**Figure 2:**
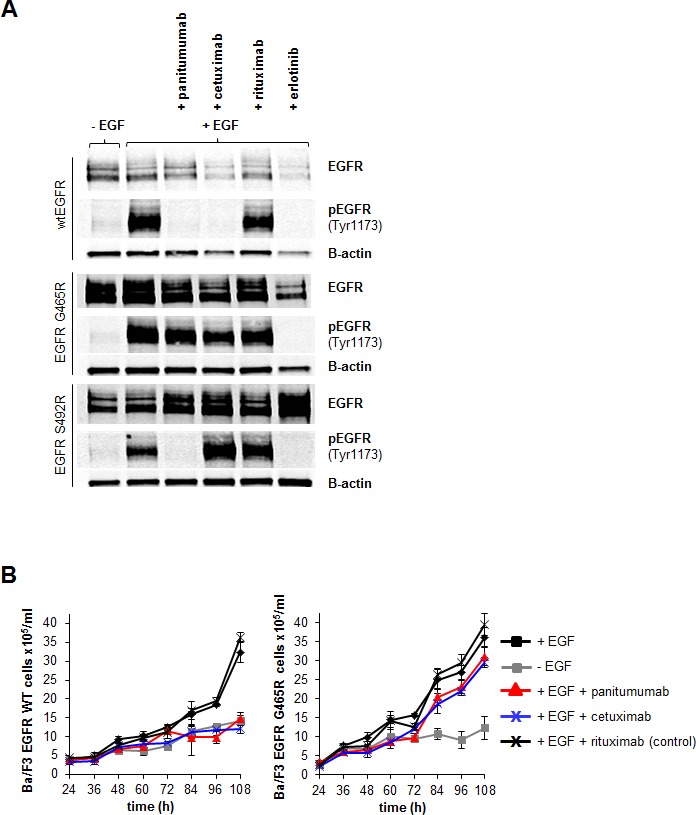
*EGFR G465R* mutation induces cross-resistance to panitumumab and cetuximab in an EGF-dependent Ba/F3 cellular model A: *EGFR* signaling in EGF-dependent Ba/F3 model. Wt and S492R or *G465R* mutant EGFR-expressing Ba/F3 cells were cultured in the presence or absence of EGF and with addition of cetuximab, panitumumab, rituximab or erlotinib. After 2 hours, cells were harvested and EGFR/pEGFR expression analyzed by western blot analysis. B: Sensitivity of *EGFR* wt or *EGFR G465R* mutant-transfected Ba/F3 cells to treatment with EGFR-targeted antibodies. Ba/F3 cells were transformed to IL-3 independence with EGFR wt or mutant constructs and subsequently cultured in the presence or absence of EGF or with EGF in combination with panitumumab, cetuximab or control antibody rituximab. The number of viable cells was determined by trypan blue exclusion every 12 hours beginning 24 hours after seeding and plotted. Data are means from triplicate experiments +/−SEM.

Next, we asked i) if receptor function was still preserved in the *EGFR G465R* mutant and ii) if the significant inhibition of antibody binding to EGFR G465R translated into resistance to panitumumab and cetuximab in a cellular model. To address this experimentally, we stably transfected murine *EGFR*-negative, IL-3-dependent Ba/F3 pro-B cells with the *EGFR* wt or mutant *G465R* and *S492R* constructs. After selection with G418, ectopic expression of wt and mutant *EGFR*in these cells conferred IL-3 independence in the presence of EGF, but not if erlotinib was added (Supplementary Figure 3). This indicated that EGF binding to the *EGFR* mutants was still preserved and receptor function intact. Stable Ba/F3 cell lines expressing wt or mutant *EGFR* were then treated with panitumumab, cetuximab, control antibody rituximab or erlotinib for 2 hours (Figure 2A). Whilst in *EGFR* wt cells cetuximab, panitumumab and erlotinib completely blocked EGFR phosphorylation, only panitumumab showed this effect in the *S492R* mutant. In the *G465R* mutant, none of the antibodies blocked EGFR phosphorylation. *EGFR* wt and *G465R* mutant Ba/F3 cells were then cultured for 108 hours in the presence of panitumumab, cetuximab or control antibody rituximab. Whilst *EGFR*wt transfected cells were sensitive to treatment with panitumumab and cetuximab, proliferation of *EGFR G465R* transfected cells was unaffected by treatment with either of these antibodies (Figure 2B). This data suggested that the *G465R* mutation, acquired under treatment with panitumumab, mediates cross-resistance to panitumumab and cetuximab by disrupting the antibody-EGFR interaction.

### Targeted *NGS* of *EGFR* and *RAS* in ctDNA of independent “liquid biopsy” patient cohort

To validate our findings, ctDNA from an independent “liquid biopsy” cohort of 22 patients treated with EGFR antibodies and 6 control cases was subjected to NGS to detect acquired *EGFR* exon 12 and *RAS* mutations (Table 3). In contrast to the “tumor tissue” cohort, these patients had clinically more advanced disease with on average 2.4 prior therapies.

**Table 3:** Clinical characteristics of the "liquid biopsy" patient cohort subjected to NGS of circulating tumor DNA.*

pat. #	category	age [years]	sex	tumor site	stage (AJCC)	KRAS status (initial)	no. of previous therapies	treatments	duration of antibody treatment [months]	response to antibody treatment	disease control at time point of plasma collection
22	panitumum abgroup	71	m	rectum	IV	wt	5	Folfoxiri/Bev. Folfiri/Pan. SM Reg. Pan-mono	6	SD/PD	PD
23		49	m	colon	III	wt	5	Ox. Folfiri/Bev. 5-FU/Ox/Pan, Pan. Lri/Mto, Folfox/Bev	7	PR/PD	n.e.
24		59	m	colon	IV	wt	4	Folfox/Bev. Pan. Cap/Bev. Fufiri/Bev	1	-	-
25		69	m	CCC	III/IV	wt	1	Cis./Gem/Pan, Gem-mono	6	SD	SD
26		46	f	colon/rectum	IV	wt	4	Cap/Ox, Muc-1, Folfiri/Pan, Pan-mono, Folfox	6	PR/PD	PD
27		63	m	CCC	IV	wt	3	Cis./Gem/Pan, Cis/Gem, Gem/Ox, Cap/Ox	8	SD	SD
28	cetuximab group	41	f	colon/rectum	IV	wt	1	Folfiri/Cet	1	-	-
29		35	f	CUP	IV	wt	1	Carbo/Pacl/Cet	1	-	-
30		59	m	rectum	IV	wt	3	Folfox, Cap/Ox, Iri/Cet, Folfiri/Cet, Cap/Ox, Cap-mono	12	SD/PD	PR
31		51	m	rectum	IV	wt	3	Folfox/Bev. Folfiri/Ram, Folfox/Cet	6	CR	CR
32		44	m	colon/rectum	I	wt	2	Folfox, Folfiri/Cet, Folfox	6	PR	PD
33		70	m	colon	IV	wt	2	Folfox/Cet, Folfox/Cet, Folfox, Folfiri, Folfiri/Cet	17	PR/PR/PR	SD
34		77	m	colon	IV	wt	1	Folfiri/Cet, Cap	6	PR	SD
35		72	m	colon	III	wt	2	Folfox, Iri/Cet	3	Pr	PD
36		52	f	rectum	IV	wt	3	Folfiri/Bev, Folfox, Iri/Cet	9	n.e.	n.e.
37		74	f	colon	III	wt	2	5-FU, Folfiri, Folfiri/Cet	5	PR	PR
38		71	f	rectum/sigma	IV	wt	5	Folfox, MGN17003, FolfiriBev, Cap/Bev, FolfiriBev, Folfox/Cet.	6	PD	PR
39		87	m	colon	IV	wt	2	5-FUFA, Iri/Cet, Cet	10	SD	SD/PD
40		72	m	sigma	III	wt	2	5-FUFA, Folfox/Cet, Folfiri/Bev	3	PR	PR
41		42	m	sigma	II	wt	1	Folfox, Folfox/Cet	4	CR	CR
42		54	f	rectum	III	wt	1	5-FU, Folfiri/Cet, Cap	6	PR	PR
43		50	f	colon	II	wt	1	Folfoxiri, Folfiri/Cet	1	-	-
44	control group (w/o EGFR targeted therapy)	70	m	colon	IV	wt	1	Cap/Ox	-	-	PD
45		53	m	colon	IV	exon 2 (n.s.)	1	Cap/Bev	-	-	SD
46		47	f	colon	n.e.	cod. 12 (G -> D)	1	Folfox/Bev, 5-FUFA/Bev	-	-	SD
47		70	f	colon	n.e.	cod. 12 (G -> V)	2	Cap. Cap/Bev. Folfiri/A fli	-	-	SD
48		74	f	colon/rectum	IV	cod. 12 (G -> V)	2	Folfox/Bev, Cap/Bev, Folfiri, tril-mono	-	-	PD
49	healthy control	29	f	-	-	-	-	-	-	-	-

* Stage refers to the stage at diagnosis; KRAS status was established by routine clinical testing at diagnosis covering exon 2 and 3 mutations; response was valuated according to recist criteria. f = female, m = male, CCC = cholangiocellular cancer, CUP = cancer of unknown primary, wt = wildtype, mut. = mutated, Pan = Panitumumab, Cet = Cetuximab, Bev= Bevacizumab, Ram = Ramucirumab, Reg = Regorafenib, FA = Folic acid, Iri = Irinotecan, Cap = Capecitabne, Gem = Gemcitabine, Mito = Mitomycin-C, Afli = Aflibercept, Cis = Cisplatin, Ox = Oxalilplatin, SM = study medication, CR = complete remission, PR = partial remission, SD = stable disease, PO = progressive disease, n.e. = not evaluated

As expected, the ctDNA analysis confirmed the *RAS* status of the control group without EGFR antibody treatment. Well in line with previous studies, we found additional acquired *RAS* mutations in about one third of patients (Table 3) [28, 29]. *RAS* mutations were exclusively found in the cetuximab patient group. However, this apparent overrepresentation did not result statistically significant by χ^2^ testing, corresponding to previously published work on acquired *RAS* mutations after both cetuximab and panitumumab treatment [28, 29].

Interestingly, 1 out of 6 panitumumab-treated patients showed evidence of the *EGFR G465R* mutation with a frequency of 7.7% (patient 25, Table 4). This patient with cholangiocellular carcinoma was unmutated for EGFR exon 12 at baseline as evidenced by NGS of his primary tumor tissue (data not shown) and had stable disease after 6 months of chemotherapy in combination with panitumumab. The blood sample was drawn 3 months after cessation of panitumumab. Of note, the previously described *S492R* mutation was not detected in our patient cohort.

**Table 4:** EGFR G465R and S492R ectodomain mutations and also KRAS and NRAS mutations after EGFR antibody treatment in circulating tumor DNA from the "liquid biopsy” patient cohort.*

pat #	category	G465R mutation	S492R mutation	KRAS (ex. 2/3/4)	NRAS (ex. 2/3/4)
22	panitumumab group	wt	wt	wt	wt
23		wt	wt	wt	wt
24		wt	wt	wt	wt
25		G465R (7.7%)	wt	wt	wt
26		wt	wt	wt	wt
27		wt	wt	wt	wt
28	cetuximab group	wt	wt	cod. 12 (GaV,3.6%)	wt
29		wt	wt	wt	wt
30		wt	wt	wt	wt
31		wt	wt	wt	wt
32		wt	wt	wt	wt
33		wt	wt	wt	wt
34		wt	wt	wt	wt
35		wt	wt	wt	wt
36		wt	wt	cod. 12 (G->V. 10.4%)	wt
37		wt	wt	cod. 12 (G->V. 5.7%)	wt
38		wt	wt	wt	wt
39		wt	wt	cod. 61 (Q->I1. 4.8%)	wt
40		wt	wt	wt	wt
41		wt	M	wt	wt
42		wt	wt	cod. 12 (G->V. 1.1%)	wt
43		wt	wt	cod. 12 (G->V. 3.2%)	wt
44	control group (w/o EGFR targeted therapy)	wt	wt	wt	wt
45		wt	wt	exon 2 (n.s.)	wt
46		wt	wt	cod. 12 (G->D. 1.1%)	wt
47		wt	wt	cod. 12 (G->V. 3.8%)	wt
48		wt	wt	cod. 12 (G->V. 25.0%)	wt
49	healthy control	wt	wt	wt	wt

* wt = wild-type;

cod. = codon; n.s. = not specified; % = percentage of reads

## DISCUSSION

Treatment and survival of patients with mCRC has improved over the past decade, largely due to the advent of new drugs, in particular targeted therapies. Around 60% of patients with *RAS* wt tumors respond to first-line chemotherapy plus EGFR-directed antibodies [2]. However, secondary resistance emerges in most patients at a median of 9 to 12 months [34]. Whilst drug resistance to EGFR-directed small molecule tyrosine kinase inhibitors has been well characterized in oncology and mutational analyses trigger therapeutic decisions in specific disease settings, resistance to monoclonal antibodies is less well understood. In the context of EGFR targeting, recent publications shed light on some of the molecular mechanisms underlying clinical resistance to cetuximab and panitumumab. These incriminated the selection of subclones with activating *RAS* mutations [28, 29] as well as an epitope-changing point mutation [31] in the *EGFR* ectodomain acquired during cetuximab treatment. The latter mechanism is of particular interest since it represents the first mutation described to confer resistance by destroying a therapeutic antibody's epitope.

These data raised several questions: How frequently do *EGFR* ectodomain mutations contribute to clinical resistance to EGFR antibody treatment in relation to acquired *RAS* mutations? How early do they occur in the course of therapy? Are other *EGFR* ectodomain mutations than the previously described *S492R* mutation acquired during EGFR inhibition (particularly during treatment with panitumumab)?

Here, we investigated *EGFR* domain III and activating *RAS* mutations in a “tumor tissue” cohort of 21 patients with gastrointestinal cancers, mainly mCRC, including 16 patients with EGFR-targeted therapy. These patients were – in their majority – clinically responsive to the antibody-containing treatment. In 1 out of 3 patients with available post-treatment samples after panitumumab-containing treatment we found a novel acquired mutation in exon 12 of the *EGFR* ectodomain. Since this mCRC patient had undergone surgery at partial remission after 3 months of FOLFOX in combination with panitumumab, we had post-treatment tumor material as well as tumor-infiltrated lymph nodes available for mutational analysis by NGS (unfortunately, no follow-up clinical data was available as this patient died due to post-surgery complications). Interestingly, the mutation was not only present in the post-treatment tumor, but also in one of the resected tumor-infiltrated lymph nodes in 3.5 and 6.8% of reads (excluding a potential sequencing artifact). The actual percentage of cells carrying the mutated receptor can, however, not directly be inferred from the percentage of reads showing the point mutation since signals of non-neoplastic cells are admixed in both materials and the sequencing of genomic DNA always yields sequences from the unmutated allele as well. Since the *G465R* mutation centrally introduces a positive charge in both epitopes, it was not surprising to find abrogation of panitumumab and cetuximab binding leading to cross-resistance to both antibodies in a cellular model. NGS of ctDNA in an independent “liquid biopsy” patient cohort showed that this mutation was present in one additional patient with cholangiocellular carcinoma. Altogether, this sums up to 2/9 patients with this resistance-mediating mutation after treatment with panitumumab. Correlating clinical outcomes of patients with the *EGFR G465* mutational status is, however, limited by the overall patient number (n=2 patients harboring the *G465R* mutation) in this study as well as the fact that these patients received antibody-chemotherapy combinations which makes it difficult to estimate the net clinical effect of the antibody. Since the mutation was found in patients who had stable disease or a partial response to chemotherapy in combination with panitumumab, we conclude that our technique may allow us to detect mutations before overt clinical resistance occurs [30, 35-38].

It remains an open question, however, if the addition of an EGFR targeting antibody to chemotherapy is entirely useless in a patient harboring a subclonal mutation or if patients may derive some benefit since the unmutated tumor cells may still be targeted. This issue needs to be prospectively addressed by future studies.

In addition to the newly discovered *EGFR* exon 12 mutation, we detected acquired *RAS* mutations in about one third of patients treated with EGFR antibodies in the “liquid biopsy” cohort, in line with previously published work [28, 29]. In contrast, sequencing performed on post-treatment tumors of the “tumor tissue” cohort did not show any acquired RAS mutations. This difference may be in part due to the fact that the “tumor tissue” cohort included less advanced disease stages than the “liquid biopsy” cohort. More importantly our data suggests that ctDNA sequencing may be more suitable for the identification of small resistant subclones since ctDNA reflects to a greater extent the genetic heterogeneity of the tumor.

Putting this investigation in relation to what other studies have shown, we most be aware that different patient cohorts were investigated. While this study was based on an unselected patient cohort with the majority of patient not considered clinically refractory to EGFR inhibition, other studies on acquired *RAS* and *EGFR S492R* mutations have been conducted in patient cohorts considered resistant to the antibody-containing regimen [28, 31]. This represents the most obvious difference between the studies and may help to explain disparities between mutational frequencies regarding the *EGFR S492R* mutation as well as acquired *RAS* mutations.

Taken together, our data shapes our understanding of epitope-changing *EGFR* mutations in gastrointestinal tumors showing that two different mutations can arise early on during EGFR-targeted treatment. These mutations are less frequently acquired as compared to *RAS* mutations and they can induce resistance or even cross-resistance depending on their localization within the panitumumab/cetuximab epitopes. Liquid biopsy strategies allow therapy monitoring and may perspectively help to guide treatment decisions in patients during EGFR targeted therapy. Validation in samples from large clinical studies of defined patient cohorts receiving chemotherapy in combination with EGFR-inhibiting antibodies are clearly warranted. We expect that these studies will help to define cut-offs for clinically significant mutational loads.

## MATERIALS AND METHODS

### Study design, patient cohorts and ethics statement

Data, tumor and blood samples of 48 patients with gastrointestinal cancer (41 CRC, 7 non-CRC) treated at our institution between February 2012 and August 2014 as well as one healthy donor were included in this prospective, longitudinal study after patients' written informed consent and approval by the ethics commission. In the first training cohort, tissue samples were analyzed pre- and post-treatment (if available) for *EGFR* and *RAS* genes as described below ("tumor tissue“ patient cohort). In the second cohort, ctDNA post-treatment was analyzed for *EGFR* and *RAS* genes in an independent set of patients ("liquid biopsy“ patient cohort).

### Targeted next-generation sequencing (NGS)

*EGFR* exons 7-13, *KRAS* exons 2/3/4 and *NRAS* exons 2/3/4 were amplified from genomic DNA isolated from paraffin embedded tumor tissue or leukocyte DNA as described in the Supplementary Detailed NGS Methods Section using the primers shown in Supplementary Table 1. NGS was performed with a median number of 27694 reads per exon per patient to detect even small tumor subclones. Sequences were aligned with the reference sequences shown in Supplementary Table 2. A schematic overview of the amplification strategies is shown in Supplementary Figure 1A.

### Generation of human *EGFR* wt and mutant constructs

cDNAs coding for the human wt EGFR ectodomain (aa1 to 645) and the human IgG1 Fc-fragment (for EGFR-Fc fusion protein expression) or the complete human wt EGFR (for membrane expression of the receptor in eukaryotic cells) were inserted into the vector pcDNA3.1(+) (Life Technologies, Carlsbad, USA). *EGFR* mutants were generated with the QuikChange XL Site-Directed Mutagenesis Kit (Agilent Technologies, Santa Clara, USA) as described [39] using individually designed oligonucleotides (Supplementary Table 3).

### Recombinant expression and purification of EGFR-Fc proteins

HEK293 cells (CRL-1573, ATCC, Manassas, USA) were transfected with *EGFR* -Fc wt and mutant constructs using Lipofectamin 2000 (Life Technologies, Carlsbad, USA). Conditioned serum-free medium was collected and EGFR-Fc proteins were purified via Protein A-Sepharose (Pierce, Appleton, USA).

### ELISA with EGFR-Fc proteins

96-well ELISA plates were coated with recombinant wt or mutant EGFR-Fc proteins. Correct expression and immobilization of EGFR-Fc proteins was assessed by ELISA using a biotinylated anti-human EGFR antibody (R&D Systems, Minneapolis, USA) and streptavidin-peroxidase conjugate (Roche, Basel, Switzerland).

To study binding of therapeutic antibodies to the fusion proteins, immobilized EGFR-Fc proteins were incubated with cetuximab (Merck, Darmstadt, Germany), panitumumab (Amgen, Thousand Oaks, USA) or rituximab (Roche, Basel, Switzerland) at 100ng/ml and detected with a biotinylated goat anti-human kappa-specific antibody (Southern Biotech, Birmingham, USA) followed by secondary detection as above.

### Transfection with *EGFR* wt and mutant constructs

*EGFR* -negative CHO cells (CCL-61, ATCC, Wesel, Germany) were chemically transfected with wt, *G465R* or *S492R EGFR* encoding vector. Ba/F3 cells (CSC-C2045, Creative Bioarray, New York, USA) kindly provided by Stefan Horn (Research Department Cell and Gene Therapy, Department of Stem Cell Transplantation, University Medical Center Hamburg-Eppendorf, Hamburg, Germany) were maintained in medium containing 10ng/ml murine IL-3 (Peprotech, Rocky Hill, USA, [40]). Electroporation-transfected cells expressing wt or mutant *EGFR* were G418-selected (1mg/ml) and subsequently cultured in the absence of IL-3 and in the presence of 10ng/ml EGF. Stable cells transformed to IL-3 independence were screened for EGFR functionality by treatment with EGF or EGF + erlotinib at 5μM (Roche, Basel, Switzerland) and subsequently used for drug-sensitivity experiments.

### Flow cytometry of transfected cells

Transfected CHO or Ba/F3 cells were stained with a polyclonal goat anti-human EGFR antibody (R&D Systems, Minneapolis, USA), panitumumab or cetuximab followed by secondary detection with FITC-labeled rabbit anti-human (Sigma-Aldrich, St.Gallen, Switzerland), or rabbit anti-goat antibodies (Dako Cytomation, Copenhagen, Denmark). Cells were analyzed on a FACS Calibur (BD Biosciences, Franklin Lakes, USA).

### *EGFR* signaling and drug-sensitivity assays

*EGFR* wt or mutant transfected Ba/F3 cells were cultured for 2 hours with or without EGF (10ng/ml), in combination with panitumumab, cetuximab, rituximab (10μg/ml), or erlotinib. Lysates were subjected to western blot analysis using the following antibodies: EGFR and phospho-EGFR (Tyr1173) (53A5) (Cell Signaling, Danvers, USA), mouse anti-β-actin (Sigma-Aldrich, St. Louis, USA), IRDye 800CW goat anti-rabbit IgG, IRDye 800CW goat anti-mouse IgG and IRDye 680RD goat anti-rabbit IgG (LI-COR Biosciences, Lincoln, USA). For drug-sensitivity assays, *EGFR* wt and *G465R* transfected Ba/F3 cells were cultured +/−EGF or with EGF/panitumumab, EGF/cetuximab or EGF/rituximab, as above. Viable cells were quantified by counting trypan blue excluding cells for 108 hours every 12 hours.

### NGS of ctDNA

ctDNA was extracted from plasma using the QIAamp Circulating Nucleic Acid Kit (Qiagen, Hilden, Germany). *EGFR* exon 12 regions surrounding positions coding for *S492* and *G465*, *KRAS* exon 2/3/4 and *NRAS* exon 2/3/4 were amplified using the primer pairs shown in Supplementary Table 1 (ctDNA). Illumina-adapter sequences / sample-specific barcodes were added as schematically shown in Supplementary Figure 1B. Sequencing (with a median number of 27 694 reads per exon per patient) as well as data analysis was performed as mentioned in the in the Supplementary Detailed NGS Methods Section.

### Statistics

Overrepresentation of *RAS* mutations in one of the two EGFR antibody-treated groups (panitumumab versus cetuximab) of both patient cohorts (“tumor tissue” and “liquid biopsy” cohort) was evaluated by chi-square-test Χ^2^ testing.

## SUPPLEMENTARY MATERIAL, FIGURES, TABLES


