# Supplementary material for: Epidermal growth factor receptor mutation mediates cross-resistance to panitumumab and cetuximab in gastrointestinal cancer
Source: Oncotarget. 2015 Mar 14;6(14):12035–47. doi: 10.18632/oncotarget.3574 (PMC4494921; doi:10.18632/oncotarget.3574)
Supplement: Supplementary file 1 [file oncotarget-06-12035-s001.pdf]

Epidermal growth factor receptor mutation mediates cross-resistance to panitumumab and cetuximab in gastrointestinal cancer

Supplementary Materials and Methods

Targeted next-generation sequencing (NGS) of EGFR, KRAS and NRAS exons

Genomic DNA was isolated from paraffin embedded tumor tissue with the QIAamp DNA Micro Kit (Qiagen, Hilden, Germany) and from leukocytes with the NucleoSpin Tissue XS kit (Macherey-Nagel, Düren, Germany). EGFR exons 7-13, KRAS exons 2/3/4 and NRAS exons 2/3/4 were amplified in two PCRs using Phusion HS II (Thermo Fisher Scientific Inc., Wilmington, USA) and adapters for hybridization and sequencing (grey) as well as barcodes for demultiplexing (blue) were attached (Supplementary Fig. 1A). All primers are shown in Supplementary Table 1. Amplicons were purified and multiplex-sequenced with a 500-cycle single indexed (8 nucleotides) paired-end run on a MiSeq Illumina sequencer. Overlapping paired reads were merged using FLASH (v1.2.6), non-overlapping reads excluded and Usearch (v6.0.307) was employed to dereplicate and cluster the merged reads. All sequences observed more then 30 times were aligned with reference EGFR, KRAS and NRAS exon sequences (Supplementary Table 2). Deviations from the germline sequence were classified as “mutations” if not identical to a known polymorphism and if present in >1% of reads.

For NGS on circulating tumor DNA (ctDNA), EGFR exon 12 was amplified as shown in Supplementary Figure 1B, KRAS exon 2/3/4 and NRAS exon 2/3/4 amplified in two PCRs as mentioned above (tumor tissue), using primers shown in Supplementary Table 1. Amplicons were purified and multiplex-sequenced with a 300-cycle single indexed (6 nucleotides) paired-end run. Data analysis was performed as above.

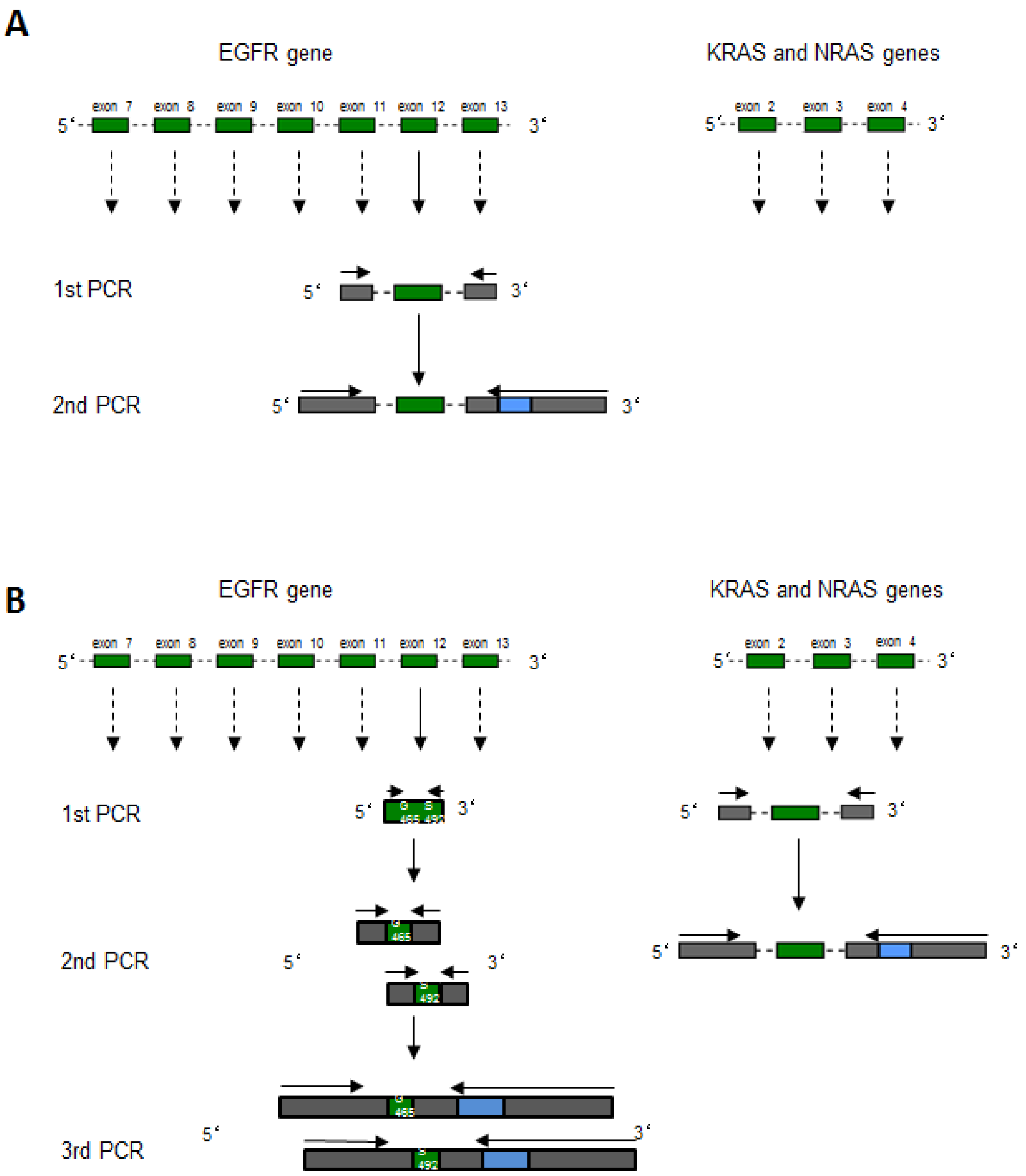

Supplementary Figure 1: PCR amplification of EGFR, KRAS and NRAS exons for targeted next generation sequencing.

A: In the "tumor tissue" patient cohort, EGFR exons 7-13, KRAS exons 2/3/4 and NRAS exons 2/3/4 (green) were amplified and Illumina-specific sequences for hybridization and sequencing (grey) as well as patient-specific barcodes (blue) were attached by a two-step PCR approach.

B: In the "liquid biopsy" patient cohort, exon 12 was amplified for the detection of the G465R and the S492R mutation by a three-step PCR approach. G = position G465; S = position S492. KRAS and NRAS exons 2/3/4 were amplified in a two-step PCR system analogous to the "tumor tissue" patient cohort shown in Suppl. Figure 1 A.

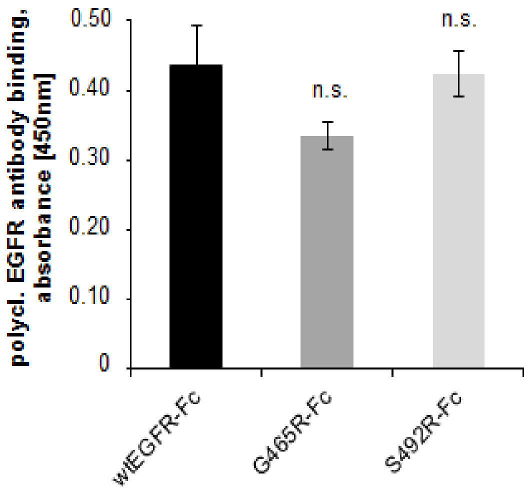

Supplementary Figure 2: Detection of EGFR-Fc wild-type and mutant proteins by ELISA.

EGFR-Fc proteins were detected using a polyclonal anti-EGFR antibody to control for correct expression of the fusion proteins. Data are means from triplicate experiments +/-SEM. n.s. = not significant (student's T-test comparing binding of polyclonal EGFR antibody to mutant versus wt EGFR-Fc)

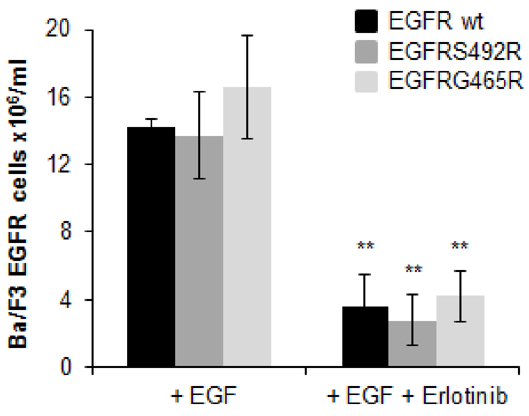

Supplementary Figure 3: Tyrosine kinase activity is preserved in EGFR ectodomain mutants.

$5x10^5$/ml wilde-type and mutant EGFR-expressing Ba/F3 cells were cultured in the presence of EGF or EGF + erlotinib (5µM). Viable cell were quantified by trypan blue exclusion after 3 days of culture. Data are means from triplicates +/- SEM. ** $p<0.01$ (student's T-test comparing viability of Ba/F3 EGFR mutant and wt transduced cells in the presence or absence of erlotinib)

Supplementary Table 1: Illumina-sequencing primers.*

template	PCR	primer name	primer sequence 5'-3'
genomic DNA from tumor biopsy	1st PCR	Seq_Exon 7_fw	ACACTCTTTCCCTACACGACGCTCTTCCGATCTccagcgtgtcctctctcct
		Seq_Exon 8_fw	ACACTCTTTCCCTACACGACGCTCTTCCGATCTcaccgtcatcaccttcctt
		Seq_Exon 9_fw	ACACTCTTTCCCTACACGACGCTCTTCCGATCTgaatacacgtctctcttatctctgcag
		Seq_Exon 10a_fw	ACACTCTTTCCCTACACGACGCTCTTCCGATCTtcaccctgttgtttgtttcag
		Seq_Exon 10b_fw	ACACTCTTTCCCTACACGACGCTCTTCCGATCTaataatcaccctgttgtttgtttcag
		Seq_Exon 11_fw	ACACTCTTTCCCTACACGACGCTCTTCCGATCTtcctacgtggtgtgtgtctga
		Seq_Exon 12_I_fw	ACACTCTTTCCCTACACGACGCTCTTCCGATCTtacattgtttttataatttttcaccac
		Seq_Exon 12_II_fw	ACACTCTTTCCCTACACGACGCTCTTCCGATCTcgctccctcaaggagataag
		Seq_Exon 13_fw	ACACTCTTTCCCTACACGACGCTCTTCCGATCTgacccactctgtctccgcag
		Seq_KRAS_Exon 2_fw	ACACTCTTTCCCTACACGACGCTCTTCCGATCTgcctgctgaaaatgactgaa
		Seq_KRAS_Exon 3_fw	ACACTCTTTCCCTACACGACGCTCTTCCGATCTccagactgtgtttctcccttc
		Seq_KRAS_Exon 4_fw	ACACTCTTTCCCTACACGACGCTCTTCCGATCTggactctgaagatgtacctatgg
		Seq_NRAS_Exon 2_fw	ACACTCTTTCCCTACACGACGCTCTTCCGATCTgttcttgctggtgtgaaatgac
		Seq_NRAS_Exon 3_fw	ACACTCTTTCCCTACACGACGCTCTTCCGATCTattgaacttccctccctccc
		Seq_NRAS_Exon 4_fw	ACACTCTTTCCCTACACGACGCTCTTCCGATCTttcccgtttttagGGAGCAGA
		Seq_Exon 7_rv	TGACTGGAGTTCAGACGTGTGCTCTTCCGATCTagacagagcgggacaaggat
		Seq_Exon 8_rv	TGACTGGAGTTCAGACGTGTGCTCTTCCGATCTctttggaggtggcatgagag
		Seq_Exon 9_rv	TGACTGGAGTTCAGACGTGTGCTCTTCCGATCTcaagcaactgaacctgtgactcac
		Seq_Exon 10a_rv	TGACTGGAGTTCAGACGTGTGCTCTTCCGATCTgggaacaggaaatatgtcgaa
		Seq_Exon 10b_rv	TGACTGGAGTTCAGACGTGTGCTCTTCCGATCTttgaaaaatatcaaaacatcagcca
		Seq_Exon 11_rv	TGACTGGAGTTCAGACGTGTGCTCTTCCGATCTcaggagctctgtgccctatc
		Seq_Exon 12_I_rv	TGACTGGAGTTCAGACGTGTGCTCTTCCGATCTtggttttctgaccggaggt
		Seq_Exon 12_II_rv	TGACTGGAGTTCAGACGTGTGCTCTTCCGATCTgacccattagaaccaactcca
		Seq_Exon 13_rv	TGACTGGAGTTCAGACGTGTGCTCTTCCGATCTcaaggggattaaagaaataacctcctac
		Seq_KRAS_Exon 2_rv	TGACTGGAGTTCAGACGTGTGCTCTTCCGATCTagaatggtcctgcaccagtaa
		Seq_KRAS_Exon 3_rv	TGACTGGAGTTCAGACGTGTGCTCTTCCGATCTtccctcattgcactgtactcc
		Seq_KRAS_Exon 4_rv	TGACTGGAGTTCAGACGTGTGCTCTTCCGATCTtcagtgttacttacctgtcttgt
		Seq_NRAS_Exon 2_rv	TGACTGGAGTTCAGACGTGTGCTCTTCCGATCTaagtggttctggattagctgga
		Seq_NRAS_Exon 3_rv	TGACTGGAGTTCAGACGTGTGCTCTTCCGATCTttggtctctcatggcactgt
		Seq_NRAS_Exon 4_rv	TGACTGGAGTTCAGACGTGTGCTCTTCCGATCTtgcaaactcttgcacaaatgc
	2nd PCR	Linker_Seq_fw	AATGATACGGCGACCACCGAGATCTACACTCTTTCCCTACACGAC
		Linker_Bar_Seq_rv	CAAGCAGAAGACGGCATACGAGAT(N_{6-7})GTGACTGGAGTTCAGACGTGTG
circulating tumor DNA	1st PCR	EGFR_Exon 12I_fw	tacattgtttttataatttttcaccac
		EGFR_Exon 12II_rv	gacccattagaaccaactcca
	2nd PCR	Seq_EGFR_G465_fw	ACACTCTTTCCCTACACGACGCTCTTCCGATCTaacatccttgggattacgct
		Seq_EGFR_S492_fw	ACACTCTTTCCCTACACGACGCTCTTCCGATCTgtgctatgcaaatacaataaactgg
		Seq_KRAS2_Exon 2_fw	ACACTCTTTCCCTACACGACGCTCTTCCGATCTgcctgctgaaaatgactgaa
		Seq_KRAS3_Exon 3_fw	ACACTCTTTCCCTACACGACGCTCTTCCGATCTccagactgtgtttctcccttc
		Seq_KRAS_Exon 4_fw	ACACTCTTTCCCTACACGACGCTCTTCCGATCTggactctgaagatgtacctatgg
		Seq_NRAS_Exon 2_fw	ACACTCTTTCCCTACACGACGCTCTTCCGATCTgttcttgctggtgtgaaatgac
		Seq_NRAS_Exon 3_fw	ACACTCTTTCCCTACACGACGCTCTTCCGATCTattgaacttccctccctccc
		Seq_NRAS_Exon 4_fw	ACACTCTTTCCCTACACGACGCTCTTCCGATCTttcccgtttttagGGAGCAGA
		Seq_EGFR_G465_rv	TGACTGGAGTTCAGACGTGTGCTCTTCCGATCTccagtttattgtatttgcatagcac
		Seq_EGFR_S492_rv	TGACTGGAGTTCAGACGTGTGCTCTTCCGATCTtgcagctgttttcacctc
		Seq_KRAS2_Exon 2_rv	TGACTGGAGTTCAGACGTGTGCTCTTCCGATCTagaatggtcctgcaccagtaa
		Seq_KRAS3_Exon 3_rv	TGACTGGAGTTCAGACGTGTGCTCTTCCGATCTtccctcattgcactgtactcc
		Seq_KRAS_Exon 4_rv	TGACTGGAGTTCAGACGTGTGCTCTTCCGATCTtcagtgttacttacctgtcttgt
		Seq_NRAS_Exon 2_rv	TGACTGGAGTTCAGACGTGTGCTCTTCCGATCTaagtggttctggattagctgga
		Seq_NRAS_Exon 3_rv	TGACTGGAGTTCAGACGTGTGCTCTTCCGATCTttggtctctcatggcactgt
		Seq_NRAS_Exon 4_rv	TGACTGGAGTTCAGACGTGTGCTCTTCCGATCTtgcaaactcttgcacaaatgc
	3rd PCR	Linker_Seq_fw	AATGATACGGCGACCACCGAGATCTACACTCTTTCCCTACACGAC
		Linker_Bar_Seq_rv	CAAGCAGAAGACGGCATACGAGAT(N_{6-7})GTGACTGGAGTTCAGACGTGTG

* Capital letters: NGS specific sequences, lower case letters: gen-specific sequences

Supplementary Table 2: EGFR and RAS reference exon sequences.

exon name	reference requences 5'-3'
EGFR exon 7	GTCTGCCGCAAATTCCGAGACGAAGCCACGTGCAAGGACACCTGCCCCCCACTCATGCTC TACAACCCCACCACGTACCAGATGGATGTGAACCCCGAGGGCAAATACAGCTTTGGTGCCA CCTGCGTGAAGAAGTGTCCCC
EGFR exon 8	GTAATTATGTGGTGACAGATCACGGCTCGTGCGTCCGAGCCTGTGGGGCCGACAGCTATG AGATGGAGGAAGACGGCGTCCGCAAGTGTAAGAAGTGCGAAGGGCCTTGCCGCAAAG
EGFR exon 9	TGTGTAACGGAATAGGTATTGGTGAATTTAAAGACTCACTCTCCATAAATGCTACGAATATTA AACACTTCAAAAACTGCACCTCCATCAGTGGCGATCTCCACATCCTGCCGGTGGCATTTAG GGG
EGFR exon 10	TGACTCCTTCACACATACTCCTCCTCTGGATCCACAGGAACTGGATATTCTGAAAACCGTAA AGGAAATCACAG
EGFR exon 11	GGTTTTTGCTGATTCAGGCTTGGCCTGAAAACAGGACGGACCTCCATGCCTTTGAGAACCT AGAAATCATACGCGGCAGGACCAAGCAACA
EGFR exon 12	TGGTCAGTTTTCTCTTGCAGTCGTCAGCCTGAACATAACATCCTTGGGATTACGCTCCCTCA AGGAGATAAGTGATGGAGATGTGATAATTTCAGGAAACAAAAATTTGTGCTATGCAAATACA ATAAACTGGAAAAAACTGTTTGGGACCTCCGGTCAGAAAACCAAAATTATAAGCAACAGAG GTGAAAACAGCTGCA
EGFR exon 13	AGGCCACAGGCCAGGTCTGCCATGCCTTGTGCTCCCCCGAGGGCTGCTGGGGCCCGGAG CCCAGGGACTGCGTCTCTTGCCGGAATGTCAGCCGAGGCAGGGAATGCGTGGACAAGTG CAACCTTCTGGAGGG
KRAS exon 2	GCCTGCTGAAAATGACTGAATATAAACTTGTGGTAGTTGGAGCTGGTGGCGTAGGCAAGAG TGCCTTGACGATACAGCTAATTCAGAATCATTTTGTGGACGAATATGATCCAACAATAGAG
KRAS exon 3	GATTCCTACAGGAAGCAAGTAGTAATTGATGGAGAAACCTGTCTCTTGGATATTCTCGACAC AGCAGGTCAAGAGGAGTACAGTGCAATGAGGGACCAGTACATGAGGACTGGGGAGGGCTT TCTTTGTGTATTTGCCATAAATAATACTAAATCATTTGAAGATATTCACCATTATAG
KRAS exon 4	GGACTCTGAAGATGTACCTATGGTCCTAGTAGGAAATAAATGTGATTTGCCTTCTAGAACAG TAGACACAAAACAGGCTCAGGACTTAGCAAGAAGTTATGGAATTCCTTTTATTGAAACATCA GCAAAGACAAGACAG
NRAS exon 2	GTTCTTGCTGGTGTGAAATGACTGAGTACAAACTGGTGGTGGTTGGAGCAGGTGGTGTTGG GAAAAGCGCACTGACAATCCAGCTAATCCAGAACCACTT
NRAS exon 3	GATTCTTACAGAAAACAAGTGGTTATAGATGGTGAAACCTGTTTGTTGGACATACTGGATAC AGCTGGACAAGAAGAGTACAGTGCCATGAGAGACCAA
NRAS exon 4	GGAGCAGATTAAGCGAGTAAAAGACTCGGATGATGTACCTATGGTGCTAGTGGGAAACAAG TGTGATTTGCCAACAAGGACAGTTGATACAAAACAAGCCCACGAACTGGCCAAGAGTTACG GGATTCCATTCATTGAAACCTCAGCCAAGACCAGACAG

Supplementary Table 3: Sequences of Mutagenesis Primers.*

primer denomination	primer sequence
**S**492**R**	GGTCAGAAAACCAAAATTATAAGAAACAGAGGTGAAAACAGC
**G**465**R**	GTGATGGAGATGTGATAATTTCAAGAAACAAAAATTTGTGCTATGC
**F**381**A**	GGGGTGACTCCGCCACACATACTCCTCC
**N**444**A**	GCAGTCGTCAGCCTGGCCATAACATCCTTGG
**S**442**A**	CAGTTTTCTCTTGCAGTCGTCGCCCTGAACATAACATCC

* All primers are designed in 5′-3′ orientation. Only forward primers are shown; corresponding reverse primer sequences are complementary reverse. Base exchanges are underlined.
